# Human mesenchymal stem/stromal cells suppress spinal inflammation in mice with contribution of pituitary adenylate cyclase-activating polypeptide (PACAP)

**DOI:** 10.1186/s12974-015-0252-5

**Published:** 2015-02-22

**Authors:** Tomomi Tsumuraya, Hirokazu Ohtaki, Dandan Song, Atsushi Sato, Jun Watanabe, Yutaka Hiraizumi, Tomoya Nakamachi, Zhifang Xu, Kenji Dohi, Hitoshi Hashimoto, Takashi Atsumi, Seiji Shioda

**Affiliations:** Department of Anatomy, Showa University School of Medicine, 1-5-8 Hatanodai, Shinagawa-ku, Tokyo 142-8555 Japan; Department of Orthopedic Surgery, Showa University Fujigaoka Hospital, 1-30 Fujigaoka, Aoba-ku, Yokohama, Kanagawa 227-8501 Japan; Department of Orthopedic Surgery, Showa University School of Medicine, 1-5-8 Hatanodai, Shinagawa-ku, Tokyo 142-8555 Japan; Laboratory of Regulatory Biology, Graduate School of Science and Engineering, University of Toyama, 3190 Gofuku, Toyama, Toyama 930-8555 Japan; Department of Emergency Medicine, Tokyo Jikei University School of Medicine, 3-25-8 Nishishinnbashi, Minato-ku, Tokyo 105-8461 Japan; Laboratory of Molecular Neuropharmacology, Graduate School of Pharmaceutical Sciences, Osaka University, 1-6 Yamadaoka, Suita, Osaka 565-0871 Japan

**Keywords:** Spinal cord injury, Human mesenchymal stem/stromal cells (hMSCs), Pituitary adenylate cyclase-activating polypeptide (PACAP), Macrophage, Microglia

## Abstract

**Background:**

Adult human mesenchymal stem/stromal cells (hMSCs) from bone marrow have been reported to exhibit beneficial effects on spinal cord injury (SCI). A neuropeptide, pituitary adenylate cyclase-activating polypeptide (PACAP) is known to decrease neuronal cell death and inflammatory response after ischemia, SCI, and other neuronal disorders. Recently, we found that expression of the gene for mouse PACAP (*Adcyap1*) was greater in animals receiving hMSCs with neural injury such as ischemia. However, the association of PACAP with hMSCs to protect nerve cells against neural injuries is still unclear.

**Methods:**

Wild-type and PACAP-gene-deficient (*Adcyap1*^*+/−*^) mice were subjected to spinal cord transection, and hMSCs (5 × 10^5^ cells) were injected into the intervertebral spinal cord on day 1 post-operation (p.o.). Locomotor activity, injury volume, retention of hMSCs, mouse and human cytokine genes (which contribute to macrophage (MΦ) and microglial activation), and *Adcyap1* were evaluated.

**Results:**

hMSCs injected into wild-type mice improved locomotor activity and injury volume compared with vehicle-treated mice. In contrast, non-viable hMSCs injected into wild-type mice, and viable hMSCs injected into *Adcyap1*^*+/−*^ mice, did not. Wild-type mice injected with hMSCs exhibited increased *Adcyap1* expression, and observed PACAP immunoreaction in neuron-like cells. Gene expression levels for IL-1, tumor necrosis factor α (TNFα), interleukin-10 (IL-10), and transforming growth factor β (TGFβ) decreased, while that for interleukin-4 (IL-4) increased, in hMSC-injected wild-type mice. In contrast, IL-1, TGFβ, and IL-4 gene expression levels were all abolished in hMSC-injected *Adcyap1*^*+/−*^ mice on day 7 post-operation. Moreover, the mice-implanted hMSCs increased an alternative activating macrophage/microglial marker, arginase activity. The human gene profile indicated that hMSCs upregulated the gene of IL-4 and growth factors which were reported to enhance *Adcyap1* expression. Finally, we demonstrated that hMSCs express human *ADCYAP1* and its receptor gene after the inflammation-related interferon-γ (IFNγ) *in vitro*.

**Conclusions:**

These results suggest that hMSCs attenuate the deleterious effects of SCI by reducing associated inflammatory responses and enhancing IL-4 production. This effect could be mediated in part by cell-cell cross-talk involving the neuropeptide PACAP.

**Electronic supplementary material:**

The online version of this article (doi:10.1186/s12974-015-0252-5) contains supplementary material, which is available to authorized users.

## Background

Every year, more than 10,000 people in the United States are victims of spinal cord injury (SCI) caused by traffic, sports, and other trauma-related accidents. While medication during the acute injury period involves the administration of large amounts of steroids and other anti-inflammatory drugs, the recovery of neurological function relies on neural plasticity and compensatory mechanisms specific to each patient. Many of these patients end up being permanently paralyzed [1,2].

Observations made on animal models suggest that a potential therapy for disorders of the central nervous system (CNS) is the administration of adult mesenchymal stem/stromal cells (MSCs) from bone marrow [3-6]. While human MSCs (hMSCs) initially attracted interest for their ability to differentiate into multiple cellular phenotypes *in vitro* and *in vivo*, part of the interest in them is due to their anti-inflammatory and immunosuppressive properties [4,7-10]. MSCs also cross-talk with host tissues to enhance protective factors and the microenvironment, inducing the release of a range of cytokines and growth factors from those tissues [4,9]. We reported that hMSCs injected into the mouse hippocampus after ischemia improved the neurological symptom, and that hMSCs increased the recipient microglia/macrophage (MΦ) to alternatively activated M2 type (AAM) [11], which are known to promote repair and regeneration after injury [12]. We also determined by microarray analysis the implanted hMSC-suppressed interferon-related genes. Recently, we reanalyzed the microarray data and found that the expression of the gene for mouse pituitary adenylate cyclase-activating polypeptide (PACAP; gene: *Adcyap1*) increased approximately ten fold in animals receiving hMSCs after ischemia compared with animals receiving the vehicle after ischemia, or hMSCs after sham-operation (see Additional file 1: Figure S1). However, no evidence has thus far been provided to demonstrate the involvement of neuropeptides in the neuroprotective properties exerted by hMSCs.

PACAP, which was first isolated from the ovine hypothalamus, belongs to the secretin/glucagon/vasoactive intestinal peptide (VIP) superfamily. PACAP exerts multiple functions through three specific receptors - PACAP receptor 1 (PAC1R) and two VIP/PACAP receptors [13,14]. Exogenous and endogenous PACAP decreases neuronal cell death after ischemia, SCI, and other neuronal disorders [15-20]. PACAP also contributes to suppress inflammatory/immune responses. *Adcyap1*-deficient mice exhibited increased deterioration in an experimental model for multiple sclerosis [21]. Severe combined immunodeficiency (SCID)-type immune-deficient mice showed decreased *Adcyap1* expression after facial nerve-crush. *Adcyap1*-deficient mice also exhibited increased levels of proinflammatory cytokines such as IL-6, tumor necrosis factor α (TNFα), and interferon-γ (IFNγ) and decreased levels of interleukin-4 (IL-4) [22]. Although a synergistic protective effect in response to co-treatment with hMSCs and PACAP after SCI has been reported [17], no evidence has been shown that hMSCs regulate the expression of PACAP.

We hypothesized here that the anti-inflammatory effect of hMSCs in response to CNS damage could involve *Adcyap1* regulation. We transplanted hMSCs into the spinal cord after a SCI in wild-type (WT) and *Adcyap1*^+/−^ mice and compared the determined neurological symptoms and mouse *Adcyap1* and *Adcyap1r1* (PAC1R gene) expression after SCI. Moreover, the effects of mouse- and human-specific cytokine gene expression to determine mechanisms underlying the anti-inflammatory action of hMSCs were also examined.

## Methods

### Animals

Wild-type C57BL/6 mice were purchased from Sankyo Lab Service Corporation (Tokyo, Japan). *Adcyap1*^+/−^ mice on a C57BL/6 background were originally provided by Dr. Hashimoto of Osaka University [23]. All mice were housed in the specific pathogen-free animal facility at Showa University and had free access to food and water. In all experiments, adult male mice (8 to 12 weeks old, weighing 17 to 25 g) were used. All experimental procedures involving animals were approved by the Institutional Animal Care and Use Committee of Showa University (#00168 and 01150).

### SCI model

The SCI mouse model was produced according to our previous report [24]. Anesthesia was induced in mice by inhalation of 4.0% sevoflurane and maintained with 3.0% sevoflurane. Under aseptic conditions, an incision was made along the midline of skin of the back, and the muscles, soft tissues, and yellow ligaments overlying the spinal column between T9 and T10 were removed. The intervertebral spinal cord between T9 and T10 was then transected with a thin-bladed razor (FEATHER, Osaka, Japan). After bleeding had stopped and coagulated blood was removed, the incision was closed and the animals were given 1.0-mL lactate Ringer’s solution (s.c., Otsuka, Tokyo, Japan) to avoid dehydration. Following recovery, foods were placed on the cage floor and the intake of the water bottle was lowered to allow for easy access. All mice were allowed to recover in a room maintained at 24°C ± 1°C during the experimental period. To support urination, the region of the lower abdomen in all mice was gently stimulated a few times a day.

### Preparation of implanted hMSCs

Frozen vials of hMSCs from bone marrow were obtained from Dr. Prockop (The Center for the Preparation and Distribution of Adult Stem Cells (http://medicine.tamhsc.edu/irm/msc-distribution.html)) under the auspices of a National Institutes of Health (NIH)/National Center for Research Resources grant (P40 RR 17 447-06). The experiments were performed with hMSCs from donor 281L [11,25]. To expand hMSCs, a frozen vial of 1.0 × 10^6^ passage 3 cells was thawed and plated at 100 cells/cm^2^ in multiple 150-mm plates (Nunclon, Thermo Fisher Scientific, Rochester, NY) with a 20-mL complete culture medium (CCM) that consisted of α-minimal essential medium (α-MEM; Invitrogen, Grand Island, NY), 20% heat-inactivated fetal bovine serum (FBS, Hyclone; Thermo Fisher Scientific), 100 units/mL penicillin, 100 μg/mL streptomycin (Invitrogen), and 2 mM L-glutamine (Invitrogen). The cultures were incubated, and the medium replaced every 3 days for approximately 8 days until cells were 70% to 80% confluent. The medium was then discarded, and the cultures plates were washed with PBS. Adherent cells were harvested with 0.25% trypsin and 1 mM EDTA (Invitrogen) for 5 min at 37°C and were resuspended at 5 × 10^5^ cells in 0.5 μL of sterile Hank’s balanced salt solution (HBSS; Invitrogen) for injection. Unviable hMSCs were prepared by repeated freezing and thawing (three times) of aliquots of these cells.

PKH26-labeled hMSCs were prepared according to instructions provided with the PKH26 Red Fluorescent Cell Linker Kit (Sigma-Aldrich, St Louis, MO). In brief, harvested hMSCs (approximately 1 × 10^7^ cells) were washed with α-MEM and centrifuged at 1,500 rpm for 7 min. The cells were then suspended for 3 min at 25°C in 1.0 mL of Diluent C with 1.0 mL of a PKH26 solution diluted 250-fold in Diluent C. Two mL of FBS was added to the suspension and incubated for 1 min at room temperature. A further 4.0 mL of CCM was added, and the suspension was centrifuged at 1,500 rpm for 6 min. After discarding the supernatant, the cells were washed three times with CCM and resuspended finally with HBSS at 5 × 10^5^ cells/mL. In a preliminary study, we confirmed that the cell suspension showed greater red fluorescence (Ex 544, Em580-10) than naive hMSCs or HBSS (Additional file 2: Figure S2). The red fluorescence of the hMSCs was also confirmed with fluorescence immunocytochemistry with CD59 (BD Bioscience) or HuNu (Chemicon) antibodies (Additional file 2: Figure S2).

### Injection of hMSCs into spinal cord

The day following surgery to invoke SCI, mice were reanesthetized by inhalation of 4.0% sevoflurane. The animals were placed face-down and a 29G-needle (HAMILTON, Reno, NV) with a 5.0-μL glass syringe (HAMILTON) was inserted directly into the intervertebral spinal cord between T10 and T11. hMSCs (5 × 10^5^ cells/μL) or HBSS were infused at a rate of 0.5 μL/min with an Ultra Micro Pump (World Precision Instruments, Sarasota, FL). After infusion, the needle was left in place for 1 min to enable the solution to diffuse into the tissue. We have shown that the fate of hMSCs was not different between immunocompetent and immunodeficient animals after ischemia [11]. Therefore, the present study did not use any immunosuppressant after cell implantation.

### Assessment of locomotor function

Motor function after SCI was assessed by using an open-field behavior test that focused on hindlimb function according to the Basso Mouse Scale (BMS) [26]. The BMS consists of an open-field locomotor rating scale, ranging from 0 (complete paralysis) to 9 (normal mobility). Briefly, individual mice were placed in the center of the open field (e.g., 50 × 50 cm^2^) with a smooth, non-slip floor and monitored for 4 min. The hindlimb movements, trunk/tail stability, and forelimb-hindlimb coordination were assessed and graded. Mice were tested daily until post-operative (p.o.) day 7. Mice with peritoneal infection, hindlimb wounds, and/or tail or foot autophagia were excluded from the study. Scoring was done by randomly numbering the mice to ensure that the investigators were not aware of the treatment groups.

### Measurement of injury volume

After anesthesia with sodium pentobarbital (50 mg/kg, i.p.), the animals were perfused transcardially on p.o. day 7 with 0.9% saline followed by 4% paraformaldehyde (PFA) in 50 mM phosphate buffer (pH 7.2) and the spinal cord removed (T7 to L1 vertebrae). Spinal cords were then post-fixed with 20% sucrose in 0.1 M phosphate buffer (pH 7.2) for two nights, and then embedded in an O.C.T. compound (Sakura Finetech, Tokyo, Japan) for subsequent preparation of frozen blocks. Five spinal cord sections (5-μm thickness) were obtained from each mouse: at the midline which included the central canal nearby to the core-injury site, and bilaterally at 30 μm and 60 μm lateral to the midline (total five sections from each mouse). The damaged area can be identified by glial fibrillary acidic protein (GFAP) immunostaining of the surrounding area, which is considered to be indicative of glial scarring [24]. The frozen sections were washed with phosphate-buffered saline (PBS) and incubated in 0.3% H_2_O_2_. The sections were blocked with 2.5% normal horse serum (NHS) in PBS for 1 h at room temperature. Subsequently, the sections were incubated overnight with rabbit anti-GFAP antibody (1:10, DAKO, Glostrup, Denmark). The sections were washed with PBS and immersed with goat anti-rabbit IgG (1:200, Santa Cruz, Santa Cruz, CA) for 2 h. They were then incubated in an avidin-biotin complex solution (Vector, Burlingame, CA) followed by diaminobenzidine (DAB; Vector) as a chromogen. Control staining involved carrying out the same steps without the incubation with the primary antibody. The injury area consisting of GFAP-immunopositive cells was measured by DP2-BSW image analysis software (Olympus, Tokyo, Japan), and the estimated injury volume was calculated by integration of the injured areas.

### Human Alu (*hAlu*) real-time PCR assays

Immediately following, and then at 7 and 14 days after injection of hMSCs, mice were anesthetized with sodium pentobarbital (50 mg/kg, i.p.) and the spinal cord was dissected. The tissue was snap frozen in liquid nitrogen and stored at −80°C until use. Genomic DNA was extracted (*n* = 4; DNeasy, Qiagen, Valencia, CA) and total DNA was assayed by UV absorbance. Real-time PCR was performed with 100 ng of target DNA, *hAlu*-specific primers, and a fluorescent probe (Model 7700; Applied Biosystems, Foster City, CA). The primers were as follows: Alu forward, 5′-CAT GGT GAA ACC CCG TCT CTA-3′; Alu reverse, 5′-GCC TCA GCC TCC CGA GTA G-3′; Probe, 5′-FAM-ATT AGC CGG GCG TGG TGG CG-TAMRA-3′. Standard curves were prepared by adding 1 × 10^2^ to 1 × 10^6^ hMSCs to samples of spinal cord from uninjured mice.

### Multiple-immunostaining

Spinal cords (T7 to L1 segment) from immediately after the injection of hMSCs and on p.o. day 7 were obtained and frozen sections prepared as described above. Microscope slides containing attached hMSCs in culture were washed three times with HBSS, fixed with 4% PFA for 15 min, and immersed in PBS containing 0.1% Tween 20 (PBST).

Tissue sections or microscope slides were washed several times with PBST and incubated in 2.5% NHS/PBST for 1 h. Subsequently, the sections were incubated overnight with primary antibodies. The sections were then rinsed with PBST and immersed with appropriate fluorescently labeled secondary antibodies for 2 h. Control staining involved carrying out the same procedures but without the incubation with primary antibodies. The primary antibodies were used as follows: rabbit anti-β2-microglobulin (B2M, 1:1000; LifeSpan Biosciences, Seattle, WA), rabbit anti-PACAP (1:1000; Peninsula Laboratories, San Carlos, CA), and rabbit anti-type1 PACAP receptor (PAC1R, 1:400). The rabbit anti-PAC1R antibody was raised by using the N-terminal residue as an antigen [15,27]. The secondary antibody used was goat anti-rabbit Alexa 488 (1:400; Invitrogen). Some sections were stained with 4,6-Diamidine-2-phenylindole dihydrochloride (DAPI, 1:10,000; Roche, Manheim, Germany) to identify cell nuclei. Fluorescence was detected using an Axio Imager optical sectioning microscope with ApoTome (Carl Zeiss, Inc.; Oberkochen, Germany).

### Isolation of RNA

Immediately after injection of hMSCs, or on p.o. days 3 or 7, mice were anesthetized with sodium pentobarbital (50 mg/kg, i.p.) and the spinal cord was dissected (T7 to L1 vertebrae). The excised tissue was snap frozen in liquid nitrogen and stored at −80°C until use. The total RNA was isolated from the cultured hMSCs or the spinal cord samples using the TRIZOL Reagent (Invitrogen, Carlsbad, CA) according to the manufacturer’s instructions. In brief, cultured hMSCs (1 × 10^6^ cells) or spinal cord tissue samples (40 mg) in 1.0 mL of TRIZOL Reagent were homogenized using a Dounce tissue grinder (WHEATON, Millville, NJ). Added to the homogenized samples was 0.2 mL of chloroform per 1 mL of TRIZOL Reagent. Following centrifugation, the aqueous phase (containing RNA) was separated from the mixture. Added to this aqueous phase was 0.5 mL of isopropyl alcohol per 1 mL of TRIZOL Reagent. After centrifugation, RNA precipitate was formed on the bottom of sample tube. The RNA precipitate was washed with 75% ethanol and dried completely at room temperature. The RNA was dissolved in RNase-free water. The purity and concentration of extracted RNA were determined spectrophotometrically (NanoDrop, Wilmington, DE). Extracted RNA was stored at −80°C until use.

### Real-time PCR

cDNA was then synthesized with a TaKaRa PrimeScript RT reagent Kit (TaKaRa BIO Inc., Shiga, Japan), using 3 μg of the total RNA. The synthesized cDNA was made up to a volume of 30 μL with sterile distilled water. Real-time PCR was performed as previously reported by our group [28], with minor modifications. Reverse transcription PCR (RT-PCR) for tumor necrosis factor α-induced protein 6 (TSG-6) was performed on a Taqman system [29], while for the others, SYBR Green was used. All human- or mouse-specific primers and probes were designed as described in Table 1.

**Table 1:** **Primers and probe to use for real-time PCR**

**Name**	**Official symbol**	**Species**	**Forward (5′ to 3′)**	**Reverse (5′ to 3′)**	**System**
PACAP	*Adcyap1*	mouse	AACCCGCTGCAAGACTTCTATGAC	TTAAGGATTTCGTGGGCGACA	Cyber green (TaKaRa)
PAC1R	*Adcyap1r1*	mouse	GGCTGTGCTGAGGCTCTACTTTG	AGGATGATGATGATGCCGATGA	
IL-1β	*Il1b*	mouse	TCCAGGATGAGGACATGAGCAC	GAACGTCACACACCAGCAGGTTA	
TNFα	*Tnf*	mouse	GTTCTATGGCCCAGACCCTCAC	GGCACCACTAGTTGGTTGTCTTTG	
IL-10	*Il10*	mouse	GACCAGCTGGACAACATACTGCTAA	GATAAGGCTTGGCAACCCAAGTAA	
TGFβ1	*Tgfb1*	mouse	GTGTGGAGCAACATGTGGAACTCTA	TTGGTTCAGCCACTGCCGTA	
IL-4	*Il4*	mouse	TCTCGAATGTACCAGGAGCCATATC	AGCACCTTGGAAGCCCTACAGA	
RPLP1	*Rplp1*	mouse	TCCGAGCTCGCTTGCATCTA	CAGATGAGGCTCCCAATGTTGA	
PACAP	*ADCYAP1*	human	GTGAGGTAAGCAAGCTCCAACAGAC	CTCGATCTGATTGCTGGGTGAA	Cyber green (TaKaRa)
PAC1R	*ADCYAPR1*	human	CTCACCACTGCCATGGTCATC	GCCCTCAGCATGAACGACAC	
NGF	*NGF*	human	AGCGTCCGGACCCAATAACA	CCTGCAGGGACATTGCTCTC	
BDNF	*BDNF*	human	GTCAAGTTGGGAGCCTGAAATAGTG	AGGATGCTGGTCCAAGTGGTG	
NT-3	*NTF3*	human	GAAACGGTACGCGGAGCATAA	GTCGGTCACCCACAGACTCTCA	
IL-4	*IL4*	human	AGCAGCTGATCCGATTCCTGA	TCCAACGTACTCTGGTTGGCTTC	
IL-10	*IL10*	human	GAGATGCCTTCAGCAGAGTGAAGA	AGTTCACATGCGCCTTGATGTC	
TGFβ1	*TGFB1*	human	GCGACTCGCCAGAGTGGTTA	GTTGATGTCCACTTGCAGTGTGTTA	
β2-microgloblin	*B2M*	human	CGGGCATTCCTGAAGCTGA	GGATGGATGAAACCCAGACACATAG	
TSG-6	*TNFAIP6*	human	AAGCAGGGTCTGGCAAATACAAGC	ATCCATCCAGCAGCACAGACATGA	Taqman (Japan Bio Service)
			probe:FAM-TTTGAAGGCGGCCATCTCGCAACTT-TAMRA		

*BDNF* brain-derived neurotrophic factor, *IL* interleikin, *NGF* nerve growth factor, *NT-3* neurotrophin 3, *PACAP* pituitary adenylate cyclase-activating polypeptide, *PAC1R* pituitary adenylate cyclase-activating polypeptide specific receptor, *RPLP1* large ribosomal protein P1, *TGFβ* transforming growth factor β, *TNFα* tumor necrosis factor α, *TSG-6* tumor necrosis factorα-induced protein 6.

### Assay for arginase activity

Arginase is a marker for AAM, and its activity was measured according to our previous report [24]. Briefly, spinal cord sections containing the T5 and L1 vertebrae from p.o. day 7 animals were removed. The tissues were homogenized with a lysis buffer (10 mM Tris-HCl (pH 7.4), 0.15 M NaCl and 1% Triton X-100, 1 mM ethylene glycol tetraacetic acid (EGTA), 50 mM NaF, 2 mM sodium orthovanadate, 10 mM sodium pyruvate, and protease inhibitor cocktail (Sigma-Aldrich)) and centrifuged at 800 × *g* for 10 min at 4°C, and the supernatant was collected. Protein concentration in the samples was determined using the BCA protein assay kit (Thermo Fisher Scientific).

The homogenate was mixed with an equal volume of pre-warmed 50 mM Tris-HCl, pH 7.5 containing 10 mM MnCl_2_ and incubated for 15 min at 55°C for activation. The mixture was then incubated in 0.25 M L-arginine for 60 min at 37°C to hydrolyze urea from L-arginine, and the reactions were stopped by adding Stop solution (H_2_SO_4_/H_3_PO_4_/H_2_O, 1:3:7). A 1% (final concentration) solution 1-phenyl-1,2-propanedione-2-oxime (ISPF, Wako, Tokyo, Japan) in ethanol was then added to the solution, which was heated at 100°C for 45 min. The reaction between urea and ISPF produced a pink color, and absorption was measured at 540 nm. Data are presented as specific activity (nmol/min/mg of protein).

### Stimulation of hMSCs with IFNγ

hMSCs were plated at 2 × 10^5^ cells/well in 6-well plates. The next day, the cells were washed twice with PBS (−) and incubated in an experimental medium (α-MEM supplemented with 1% FBS, 100 units/mL penicillin, 100 μg/mL streptomycin, and 2 mM L-glutamine). Then the cells (*n* = 3 plates for each phenotype) were exposed to a recombinant mouse IFNγ (10 ng/mL, PeproTech, Rocky Hill, NJ) or vehicle. Forty-eight hours later, the cells were collected and stored at −30°C until analysis.

### Stimulation of MΦ-differentiated U937 with LPS and RNA isolation

Human monocyte-like cell line U937 was obtained from the RIKEN Cell Bank (Tsukuba, Japan). For routine maintenance, cells were cultured in RPMI 1640 medium (Invitrogen) supplemented with 10% FBS and 1.0% penicillin/streptomycin in 5% CO_2_ at 37°C in a humidified chamber. Cell concentrations were maintained between 2 × 10^5^ and 2 × 10^6^ cells/mL. For differentiation into MΦ, 5 × 10^5^ cells were placed into the wells of a 12-well plate and treated with 20 nM phorbol myristate acetate (PMA, Sigma-Aldrich) for 24 h to induce the MΦ-like adherent phenotype. Subsequently, the medium was replaced with fresh RPMI 1640 medium and cells cultured for a further 48 h. PMA-induced U937 cells were stimulated with 0.1 μg/mL of lipopolysaccharide (LPS, Sigma-Aldrich) for 24 hours as a positive control.

### RT-PCR for human PACAP and PAC1R

RT-PCR was performed as reported previously [30]. Briefly, the total RNA was extracted from cell pellets (hMSCs, hMSCs stimulated with IFNγ, MΦ-like differentiated U937, MΦ-like differentiated U937 stimulated with LPS) by TRIZOL reagent (Invitrogen) according to the manufacturer’s instructions. The purity and concentration of extracted RNA were determined spectrophotometrically (NanoDrop, Wilmington, DE). The cDNA was then synthesized with an AffinifyScript QPCR cDNA Synthesis Kit (Stratagene, Agilent Technologies, La Jolla, CA), using 1 μg of the total RNA. The synthesized cDNA was made up to a volume of 50 μL with sterile water supplied with the kit. The reaction mixture contained 0.6 μL of the first-strand cDNA, 7 pmols of each primer set and 6.0 μL of the Emerald Amp PCR Master Mix (2X premix) (TaKaRa) in a total volume of 12 μL. The primers were as follows: hPACAP forward, 5′-GAAACAAATGGCTGTCAAGAAA-3′; hPACAP reverse, 5′- TCTGTGCATTCTCTAGTGCTTTG-3′; hPACAPR1forward, 5′-GTTACTTCGCTGTGGACTTCAA-3′; hPACAPR1 reverse, 5′-GGACCAGTACCAAAACAAGGAG-3′; hGAPDH forward, 5′-GGTGGTCTCCTCTGACTTCAAC-3′; hGAPDH reverse, 5′-GTCTACATGGCAACTGTGAGGA-3′. Thermal-cycling parameters were set as follows: 97°C for 5 min for an initial denaturation, then a cycling regime of 40 cycles at 95°C for 45 s, 60°C for 45 s, and 72°C for 1 min. At the end of the final cycle, an additional extension step was carried out for 10 min at 72°C. Three microliters of each reaction mixture were loaded for 1.6% agarose gel electrophoresis and bands were visualized with ethidium bromide.

### Statistical analysis

Each mouse was assigned a random number, and all data were collected and analyzed without investigator knowledge of group identities. Data are expressed as mean ± SEM for *in vivo* experiments and as mean ± SD for *in vitro* experiments. Statistical comparisons were made by Student’s *t*-test for two groups and by one-way ANOVA following non-parametric multiple comparison as indicated in each figure legend. A value of *P* < 0.05 was considered to indicate statistical significance.

## Results

### Injection of hMSCs improved locomotor activity and suppressed SCI-related damage

**Figure 1:**
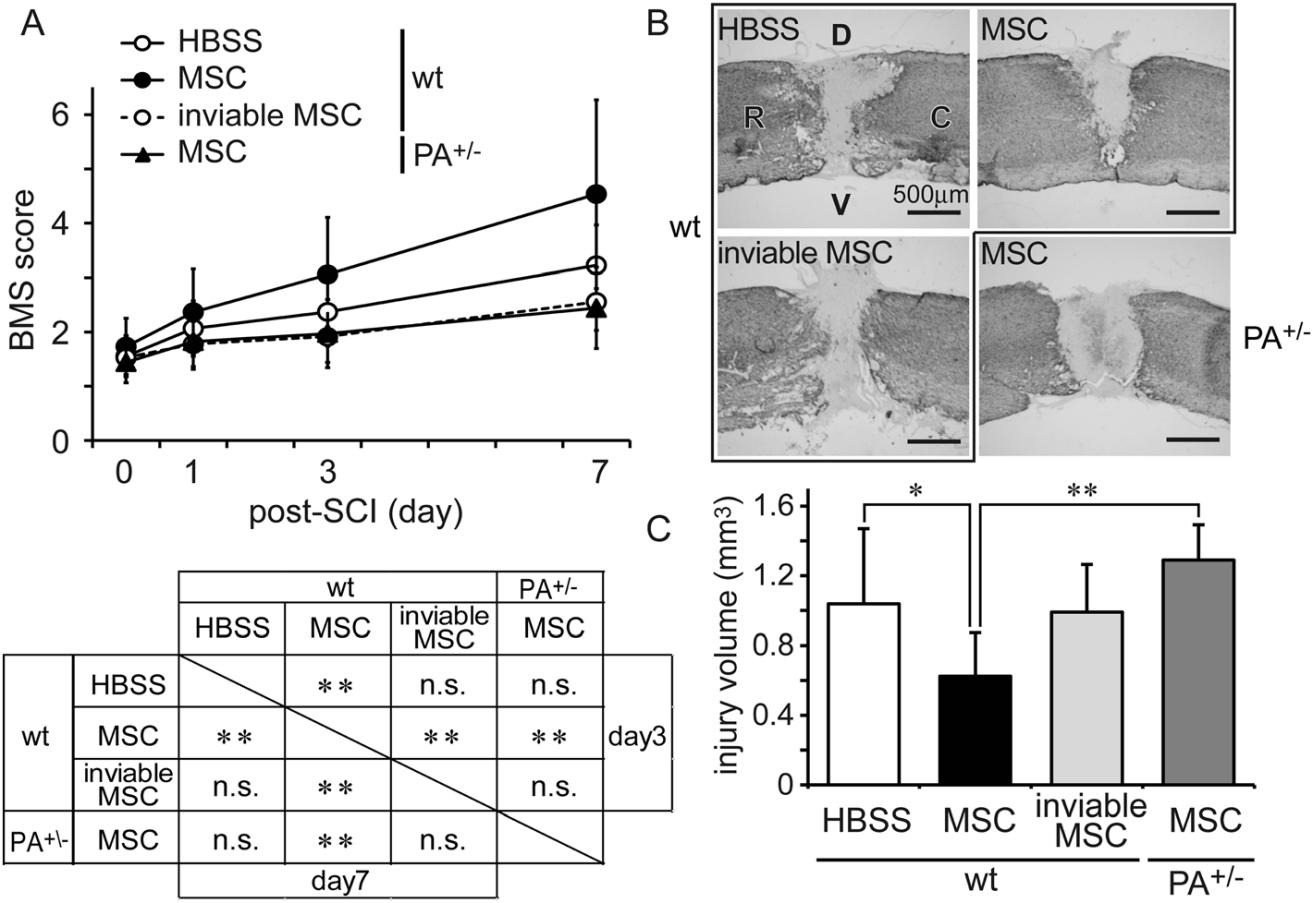
**Injection of viable hMSCs suppresses SCI symptoms in wild-type (WT) mice. (A)** Motor function determined by BMS improved significantly after injection of viable hMSCs (filled circles, *n* = 67) compared with that of HBSS (open circles with solid line, *n* = 47) in WT mice. This improvement was diminished when unviable hMSCs were injected into WT mice (open circles with dashed line, *n* = 18) or when viable hMSCs were injected into *Adcyap1* ^*+/−*^ mice (filled triangles, *n* = 17). Data are expressed as mean ± SEM. **P < 0.01 (Tukey *post hoc t-*test). **(B)** Typical images of lesion site after SCI. Injection of viable hMSCs (*right upper*) into WT mice decreased the lesion area compared with that in HBSS-treated control (*left upper*). Unviable hMSCs injected into WT mice (*left bottom*) and viable hMSCs injected into *Adcyap1* ^+/−^ mice (*right bottom*) did not reduce the lesion size. R: rostral, C: caudal, D: dorsal, V: ventral. Scale bar is 500 μm. **(C)** Quantification of GFAP-unstained injury volume at 7 days. Data are expressed as mean ± SEM. *P < 0.05, **P < 0.01 (Tukey *post hoc t-*test). *BMS* Basso Mouse Scale, *HBSS* Hank’s balanced salt solution, *MSC* mesenchymal stem/stromal cell, *n.s.* no significant, *PA* ^*+/−*^ *Adcyap1* (PACAP gene) heterozygous mice, *SCI* spinal cord injury, *wt* wild-type.

We first determined locomotor activity and lesion size after injecting hMSCs into the spinal cord. This intervention induced severe locomotor deficits (BMS of around 1.5 points; see the ‘Methods’ section) [26] within a few hours. On p.o. day 1, hMSCs (5 × 10^5^ cells/0.5 μL) were injected into the intervertebral spinal cord one vertebra rostral to the injury site in WT or *Adcyap1*^+/−^ mice. Another set of mice were injected with repeat freeze-thawed unviable hMSCs. WT mice implanted with viable hMSCs (hMSC/WT mice) exhibited significantly improved BMS on p.o. days 3 and 7 compared with vehicle-treated WT one (HBSS/WT mice). However, WT mice implanted with unviable hMSCs (unviable hMSC/WT mice) and *Adcyap1*^+/−^ mice implanted with viable cells (hMSC/*Adcyap1*^+/−^ mice) had significantly lower BMS scores than the hMSC/WT mice (Figure 1A).

We next compared lesion areas on p.o. day 7. hMSC/WT mice exhibited significantly reduced lesion area compared with HBSS/WT mice, whereas unviable hMSC/WT and hMSC/*Adcyap1*^+/−^ mice did not show any improvement (Figure 1B, C).

### Fate of hMSCs in the spinal cord

**Figure 2:**
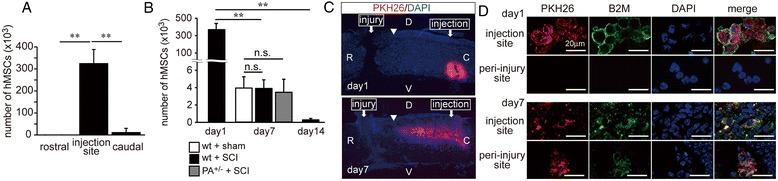
**Distribution of implanted hMSCs in the spinal cord and distribution of PKH26-labeled hMSCs after hMSC injection, with higher magnification images.** Distribution of implanted hMSCs in the spinal cord **(A, B)**. **(A)** Real-time PCR assays for *hAlu* after injection of hMSCs (5 × 10^5^ cells) into spinal cord (*n* = 4). Data are expressed as mean ± SEM. **P < 0.001 (Dunnet *post hoc t-*test vs injection site). **(B)** Temporal profile of hMSC survival after injection in animals with or without SCI. The survival of hMSCs in wild-type (WT) mice with SCI decreased drastically with time. Data are expressed as mean ± SEM. **P < 0.01 (Dunnet *post hoc t-*test). **(C)** Distribution of PKH26-labeled hMSCs (*red*) after hMSC injection. Within 10 min of injection (day 1), the cells were observed as a cluster in the injection site (*injection*). At 7 days after SCI, the cells are seen to have migrated along the spinal cord toward the injury site (*injury*) and detected the PKH26 signals in peri-injury site (*arrowhead*). Blue: DAPI counterstaining. R: rostral, C: caudal, D: dorsal, V: ventral. **(D)** Higher magnification images of **(C)** with human B2M staining (*green*) shows co-labeling with PKH26-stained cells. One day after SCI and immediately after hMSC injection, no PKH26 signals can be observed in the peri-injury site. However, PKH26-red signals merged with B2M-positive reactions (*green*) can be observed on the peri-injury site on post-operative day 7. *B2M* β2-microglobulin, *DAPI* 4,6-Diamidine-2-phenylindole dihydrochloride, *hMSC* human MSC, *n.s.* no significant, *PA* ^*+/−*^ *Adcyap1* (PACAP gene) heterozygous mice, *SCI* spinal cord injury, *wt* wild-type.

We next observed the fate of hMSCs with human *hAlu* quantitative real-time PCR and estimated number of hMSCs after implantation. Within 10 min of injection of hMSCs, 326,058 ± 62,153 hMSCs cells (>65%) were counted from the injection site, 424 ± 173 cells were measured from the rostral site, and 12,322 ± 9,126 cells from the caudal site (Figure 2A). The number of hMSCs decreased rapidly to 3,879 ± 2,704 and 250 ± 169 (1/100 and 1/10,000 immediately after implantation) on p.o. day 7 and 14, respectively. The estimate numbers of hMSCs in the spinal cord from the non-injured WT and injured *Adcyap1*^+/−^ mice on p.o. day 7 after hMSCs treatment were not different from that in the injured WT mice (Figure 2B).

Migration of hMSCs toward the injection site was confirmed by PKH26-fluorescence labeling. On the day of cell injection (p.o. day 1), red fluorescence was clustered around the injective site and merged with human B2M immunoreactivity, but was not observed at the injury site. The fluorescence migrated toward the injury site and merged with the B2M immunoreactivity in both of the injection and peri-injury sites by p.o. day 7 (Figure 2C, D).

### *Adcyap1* and *Adcyap1r1* expression were induced by hMSCs

**Figure 3:**
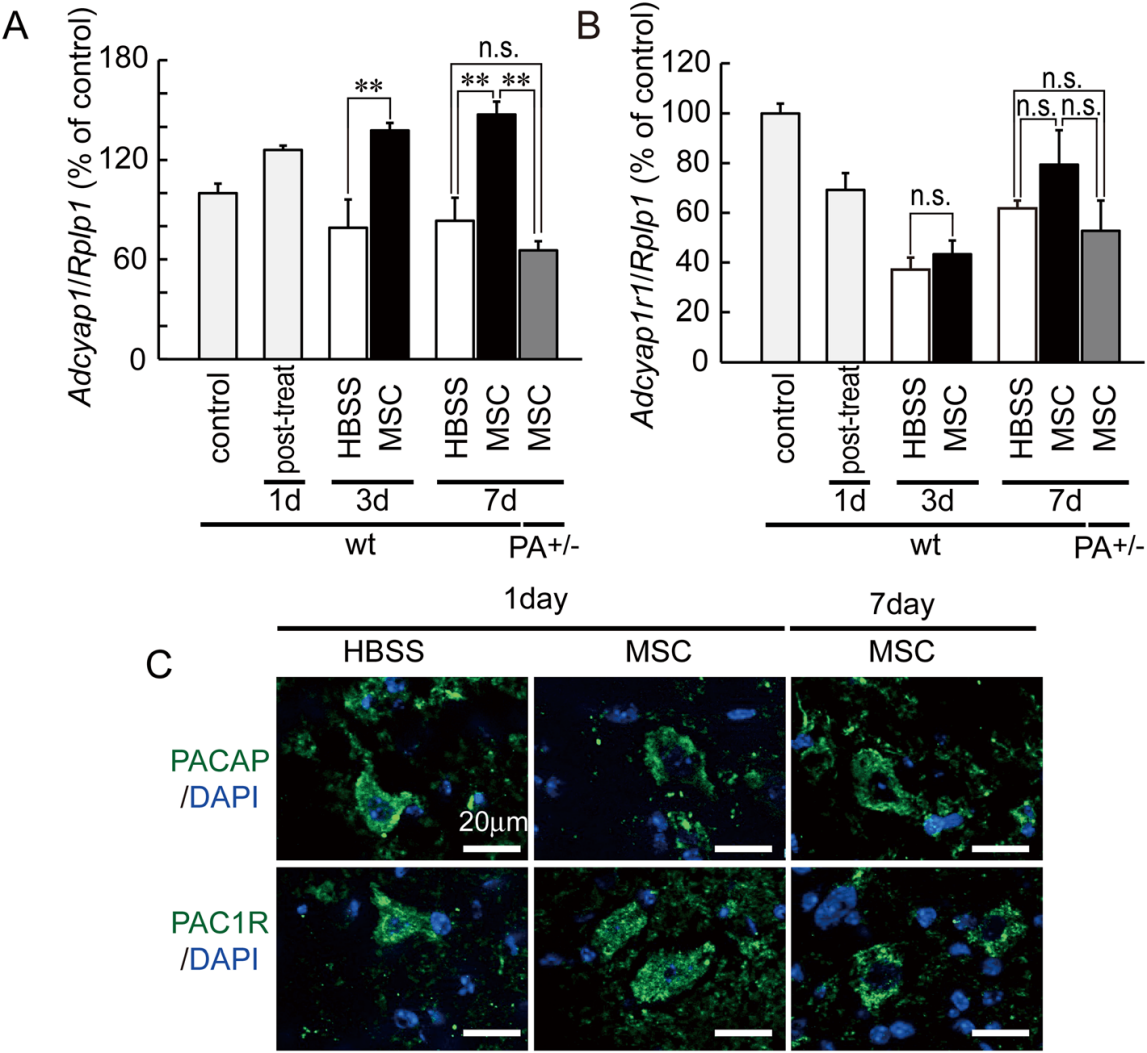
**Implanted hMSCs induced increased mouse** ***Adcyap1*** **expression after SCI.** Mouse *Adcyap1* **(A)** and *Adcyap1r1* **(B)** expression levels in spinal cord after injury and in the presence and absence of injected hMSCs. **(A)** Although *Adcyap1* expression in HBSS-treated animals decreases after SCI, in hMSC-treated animals it increases significantly. Gene expression levels are similar between HBSS-treated WT mice and hMSC-treated *Adcyap1* ^+/−^ mice at 7 days. (B) *Adcyap1r1* expression is similar for all experimental groups. *Rplp1* is the large ribosomal protein P1 as a house keeping gene. Data are expressed as mean ± SEM. **P < 0.01 (Tukey *post hoc* test). **(C)** Multiple-immunostaining of PACAP or PAC1R (*green*) and nuclei (*blue*; DAPI). *DAPI* 4,6-Diamidine-2-phenylindole dihydrochloride, *HBSS* Hank’s balanced salt solution, *MSC* mesenchymal stem/stromal cell, *PA* ^*+/−*^ *Adcyap1* (PACAP gene) heterozygous mice, *PACAP* pituitary adenylate cyclase-activating polypeptide, *PAC1R* pituitary adenylate cyclase-activating polypeptide specific receptor, *wt* wild-type.

We reported previously that *Adcyap1*^+/−^ mice increased lesion size to compare with the WT mice after brain ischemia [15] and contusion-induced SCI [19]. Therefore, failure of hMSCs to induce tissue protection in *Adcyap1*^+/−^ mice could have been due to the deletion of *Adcyap1*. For this reason, we next examined mouse *Adcyap1* and *Adcyap1r1* induction after the injection of hMSCs (Figure 3A, B). In HBSS/WT, *Adcyap1* expression in the spinal cord was temporarily increased on p.o. day 1 and then decreased. *Adcyap1* expression in hMSC/WT mice was significantly greater than that in HBSS/WT and hMSC/*Adcyap1*^+/−^ mice. *Adcyap1r1* expression levels in the spinal cord did not differ among any of the experimental groups. PACAP and PAC1R immunoreactivities were mainly observed in neuron-like cells (Figure 3C).

### Alteration of mouse cytokine profiles in spinal cords injected with hMSCs

Several factors have been reported concerning hMSCs’ capacity to decrease inflammation via MΦ; hMSCs increase AAM via the induction of IL-4 [11], they suppress classical activating MΦ (CAM) which are induced by IFNγ [25,29], and they increase deactivating MΦ (DAM) which are induced by interleukin-10 (IL-10) [10].

**Figure 4:**
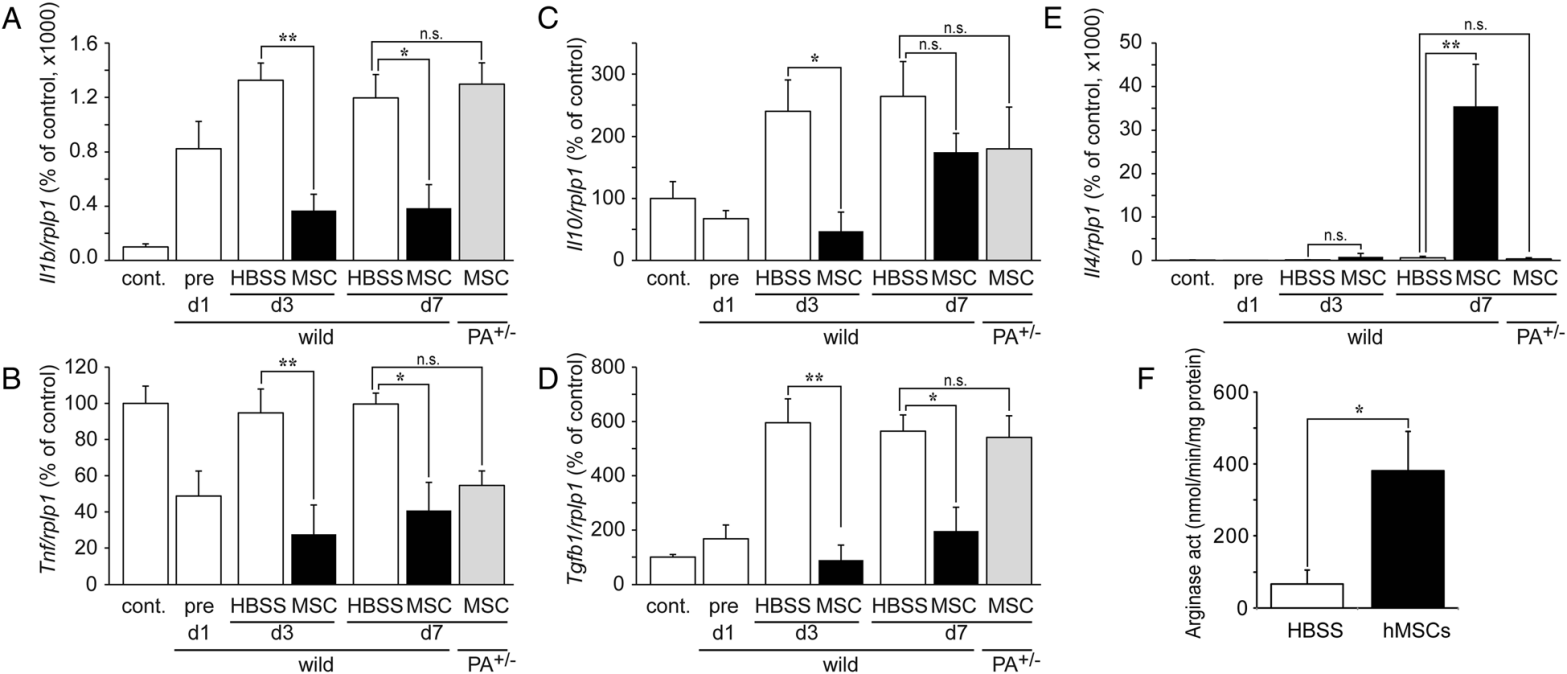
**Implantation of hMSCs reduced mouse pro-inflammatory gene profiles and favored the M2-type alternative activating microglial/Mϕ (AAM) environment.** Mouse cytokine gene expressions were determined in spinal cords after injury. hMSC-treated wild-type (wild) mice (*black*) manifested suppressed levels of mouse proinflammatory cytokines gene expression such as IL-1β (*Il1b*; **(A)**) and TNFα (*Tnf*; **(B)**) compared with HBSS-treated mice (*white*). The cell treatment also decreased IL-10 (*Il10*; **(C)**) and TGFβ (*Tgfb1*; **(D)**) levels. However, hMSC-treated wild-type mice exhibited increased levels of gene expression for anti-inflammatory cytokine such as IL-4 (*Il4*; **(E)**). For *Il1b*, *Tnf*, *Tgfb1*, and *Il4*, these were diminished in hMSC-treated *Adcyap1* ^+/−^ (PA^+/−^) mice (*gray*) 7 days after SCI. Cont means intact spinal cord in wild-type mice. Data show mean ± SE (*n* = 8). *P < 0.05, **P < 0.01, ***P < 0.001 (Tukey *post hoc* test). Injection of hMSCs significantly increased arginase activity 7 days after SCI **(F)**. Data show mean ± SE (*n* = 7). *P < 0.05 (*Student’s t*-test). *HBSS* Hank’s balanced salt solution, *MSC* mesenchymal stem/stromal cell, *n.s.* no significant, *PA* ^*+/−*^ *Adcyap1* (PACAP gene) heterozygous mice.

We then assayed expression levels of mouse inflammatory cytokines in the spinal cord. The mouse interleukin-1β (IL-1β) gene (*Il1b*) and TNFα gene (*Tnf*) in HBSS/WT mice showed greater expression after SCI. hMSC/WT mice suppressed significantly the expression levels. However, in hMSCs/*Adcyap1*^+/−^ mice, the level of *Il1b* expressed was similar to that in the HBSS/WT. (Figure 4A, B). Expression levels for the IL-10 (*Il10*) and TGFβ1 (*Tgfb1*) genes increased in the HBSS/WT mice after SCI and decreased significantly in the hMSC/WT mice. hMSC/*Adcyap1*^*+/−*^ mice exhibited a similar level of expression of *Tgfb1* as that seen in HBSS/WT, while *Il10* expression was not modified (Figure 4C, D). Low expression levels of the IL-4 gene (*Il4*) were seen in HBSS/WT on p.o. day 7; in contrast, drastically increased levels were seen in the hMSC/WT mice, while no expression was seen in the hMSC/*Adcyap1*^*+/−*^ mice (Figure 4E). We then assayed for arginase activity, an AAM marker, in WT mice with or without injected hMSCs and demonstrated that the activity was significantly increased in the hMSC/WT mice compared with the HBSS/WT mice (Figure 4F).

### Genetic profile of hMSCs in the spinal cord

PACAP has been reported to induce the gene expression of some growth factors and Th2-type cytokines. We next determined selected human gene expression levels according to previous studies [22,31,32].

**Figure 5:**
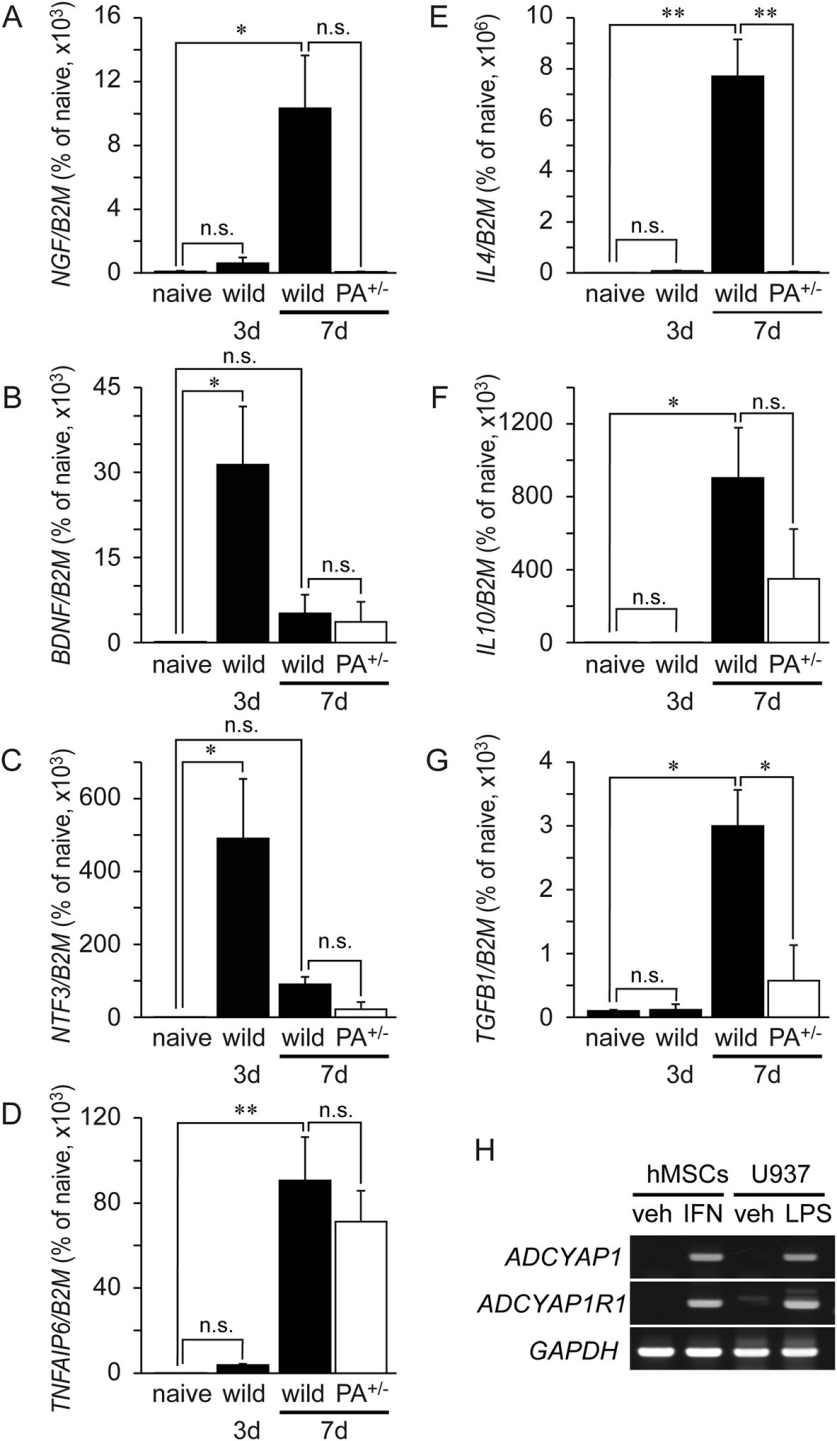
**Human gene profiles after injection of hMSCs into injured spinal cord.** Real-time RT-PCR assays with human-specific primers of naive hMSCs and injured spinal cord after injection of hMSCs into wild-type (wild) and *Adcyap1* ^+/−^ (PA^+/−^) mice. Human BDNF (*BDNF*; **(B)**) and NT3 (*NTF3*; **(C)**) increased 3 days after SCI. Human NGF (*NGF*; **(A)**), TSG-6 (*TNFAIP6*; **(D)**), IL-4 (*IL4*; **(E)**), IL-10 (*IL10*; **(F)**), and TGFβ (*TGFB1*; **(G)**) increased 7 days after SCI. Injection of hMSCs into *Adcyap1* ^+/−^ mice with SCI resulted in a decrease of human *IL4* and *TGFB1* expression. Naive means aliquot of cultured hMSCs before transplantation. Data are expressed as mean ± SE (*n* = 8). *P < 0.05, **P < 0.01 (Tukey *post hoc* test). Human *ADCYAP1* and *ADCYAP1R1* expression in hMSCs stimulated with IFNγ (*in vitro*) **(H)**. hMSCs exposed to IFNγ for 48 h showed increased *ADCYAP1* and *ADCYAP1R1* expression. MΦ-like differentiated U937 was used as a positive control for the gene expression. *BDNF* brain-derived neurotrophic factor, *B2M* β2-microglobulin, *NGF* nerve growth factor, *n.s.* no significant, *PA* ^*+/−*^ *Adcyap1* (PACAP gene) heterozygous mice.

Gene expression levels of growth factors such as the nerve growth factor (*NGF*), brain-derived neurotrophic factor (*BDNF*), and neurotrophin 3 (*NTF3*) were temporarily but significantly increased in the hMSC/WT mice compared with cultured hMSCs on p.o. days 3 or 7 (Figure 5A, B, C). The expression of genes for Th2-type cytokines such as *IL4*, *IL10*, and *TGFB1* was also significantly increased on p.o. day 7 (Figure 5E, F, G). Moreover, we assayed TSG-6 (*TNFAIP6*) as well. TSG-6 from hMSCs has been reported to decrease inflammatory responses in peritoneal and traumatic brain injury [29,33] and was found here to be significantly increased in the spinal cord of hMSC/WT on p.o. day 7 compared to that in HBSS/WT (Figure 5D).

In the hMSCs/*Adcyap1*^*+/−*^ mice, while gene expressions for growth factors and IL10 tended to decrease in *Adcyap1*^*+/−*^ mice, no significant differences were found to compare with the WT mice (Figure 5A, B, C, F). *TNFAIP6* was not altered in the *Adcyap1*^*+/−*^ mice (Figure 5D); however, the *IL4* and *TGFB1* expressions were significantly down-regulated (Figure 5E, G).

The possibility exists that PACAP acts directly on hMSCs, most likely via PAC1R. Although we initially assayed the *ADCYAP1* and *ADCYAP1R1* gene expressions in the spinal cord of hMSC/WT, these were not detected (data not shown). Expression of these genes was then examined *in vitro* on hMSC cultures where we added 10 ng/mL IFNγ (or vehicle alone for control) to mimic inflammatory conditions (Figure 5H). IFNγ-treated hMSCs exhibited increased *ADCYAP1* and *ADCYAP1R1* at 24 h, whereas the vehicle alone did not express the genes.

## Discussions

We demonstrated here that when hMSCs were injected on p.o. day 1 into the spinal cord of WT mice, subsequent improvements were seen in various parameters which suggested to reduce SCI, but inviable hMSCs did not exert these effects. We observed also that these findings could not be reproduced in *Adcyap1*^+/−^ mice. Because the retention of hMSCs was no different between the hMSC/WT and hMSC/*Adcyap1*^+/−^ mice, we considered that the beneficial effect of hMSCs was due to cross-talk between the hMSCs and recipient tissues involving the action of PACAP. PACAP is a well-documented neuropeptide that suppresses cell death in ischemia, SCI, and other CNS disorders [15-17,19-21]. We previously demonstrated the exacerbation of cell death in *Adcyap1*^+/−^ mice to compare with a WT mouse after ischemia and SCI [15,19]. In the present study, we however could not see significant difference in the neural damage in the HBSS-injected animals both for the WT and *Adcyap1*^+/−^ mice. Therefore, it was considered that it may be only competing between an increase of the cell death in *Adcyap1*^*+/−*^ and suppression of the cell death by hMSCs. Then, we examined the expression of recipient mouse *Adcyap1* and *Adcyap1r1* in the spinal cord after implanted hMSCs and demonstrated clearly that hMSC transplantation exhibited an increase of mouse *Adcyap1*. These findings strongly suggest that hMSCs may contribute to neuroprotection with PACAP induction.

To determine how hMSCs induced *Adcyap1* expression, we studied recipient mouse and donor hMSC gene expressions. So far, it is reported that PACAP has been induced by cyclic AMP, amyloid β-protein, CREB, progesterone, growth factors such as BDNF and NGF, and PACAP itself *in vitro* and *in vivo* experiments [31,32,34]. The expression of *Adcyap1* might be also influenced by immune/inflammatory stimuli, in particular IL-4-related stimuli, given the decreased expression seen in SCID mice after nerve injury [22]. In the present study, we observed that the hMSCs after transplantation increased growth factors such as NGF, BDNF, and NTF3. These have been suggested to increase Adcyap1 expression [31,32,34] and are released from hMSCs after implantation [35]. hMSCs increased an expression of anti-inflammatory cytokines such as *IL4* and *IL10*, and *TGFB1* as well. Indeed, mouse *Il4* and an AAM marker, arginase activity, were greater in the hMSC/WT of the spinal cord. It also suggests that microenvironment PACAP expressions are produced in the spinal cord after hMSCs implantation.

We next investigated how hMSCs suppressed SCI and how PACAP associated the effect. Several studies including ours suggested that hMSCs modulated the inflammatory response in recipient tissues, in particular that of microglia/MΦ activation. Microglia/MΦ show different types of activation - CAM, AAM, and DAM - depending on the cytokine stimuli involved [12,24]. After hMSCs implantation, a mouse proinflammatory cytokine gene such as *Il1b* and *Tnf* is significantly suppressed, suggesting that hMSCs modulated CAM in the spinal cord. We have reported that a hMSC-mixed culture with mouse microglial cells under IFNγ stimuli decreased the level of nitric oxide in a hMSC-number-dependent fashion [25]. We reported also that hMSCs increased the expression of *TNFAIP6* in the present study and the traumatic brain injury model [33]. *TNFAIP6* (also known as TSG-6) is a candidate factor to be involved in hMSCs’ anti-inflammation. *TNFAIP6* increased from hMSCs after the implantation in the cardiac infarction, global ischemia, and peritoneal inflammation [11,29,36] and suppressed TNFα [29,36,37]. Conversely, hMSC implantation increased both human and mouse IL-4 gene expression and arginase activity in the recipient tissue, suggesting that hMSCs increased AAM [12,38,39] which consisted with global ischemia [11]. It has been reported that hMSCs increased CAM mediated by IL-10 and transforming growth factor β (TGFβ) [12] in a hippocampal organotypic culture [40] and sepsis mouse [10].

However, our observation showed a decrease in the *Il10* and *Tgfb1* expression, probably due to a decrease of proinflammatory cytokine. Like these, we suggest that hMSCs decreased CAM and inflammation and induced a resolution by AAM. Our results interestingly suggested that hMSCs modulated mouse cytokine profile at least via two different pathways: PACAP-dependent and PACAP-independent pathways. *Il1b*, *Il4*, and *Tgfb1*, and part of *Tnf*, were abolished in *Adcyap1*^+/−^ mice, suggesting PACAP worked as a mediator between recipient tissue and donor hMSCs. On the other hand, *Il10* and most of *Tnf* were independent with endogenous PACAP. We reported previously that *Il4* and AAM decreased in *Il1a*- and *Il1b*-deficient mice after SCI although the injury area was suppressed in the deficient mice. Like these, the cytokines form a complicated network during the disease [24].

We hypothesized first that PACAP acts downstream of hMSCs and that it does not influence the human gene profile. However, our results indicated that the endogenous mouse PACAP might modulate the hMSCs’ function because hMSC/*Adcyap1*^+/−^ mice influenced human gene expression. For example, *IL4* and *TGFB1* were influenced by PACAP, whereas *TNFAIP6* and *IL10* were not the same as mice gene profiles. These indicated that recipient tissue communicated between hMSCs and PACAP or a factor mediated by PACAP. To the present time, no studies have reported that hMSCs express PAC1R. We firstly examined human *ADCYAP1* or *ADCYAP1R1* in the implanted spinal cord. However, we failed to detect the gene expression. Then, we examined the *in vitro* study and observed slight increases of them after IFNγ stimulation *in vitro*. This result suggests that hMSCs could express PAC1R in response to inflammatory conditions, thus enabling hMSCs to communicate with recipient tissues via autocrine and paracrine processes partially mediated by PAC1R. The contribution of human *ADCYAP1 in vivo* is still unclear because we could not detect human *ADCYAP1* in the spinal cord. Using RNA interference or other techniques, we need to clarify how much human PACAP contributed to the communication. This synergistic cross-talk may enhance anti-inflammatory processes and give rise to an AAM environment. Further studies are needed to clarify the central player(s) in this communication and the complicated cytokine network.

## Conclusions

**Figure 6:**
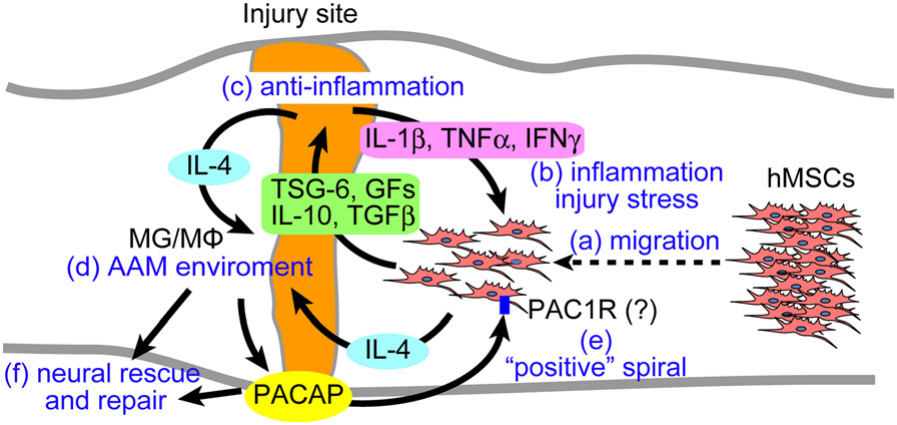
**Schematic illustration of the putative neuroprotective mechanism underlying the effect of cross-talk between hMSCs and PACAP in animals subjected to SCI (see the ‘Discussions’ section)** *AAM* alternatively activated (activating) macrophage, *GFs* growth factors, *hMSCs* human MSCs, *IFNγ* interferron-γ, *IL* interleikin, *MG/MΦ* microglia and/or macrophage, *PACAP* pituitary adenylate cyclase-activating polypeptide, *PAC1R* pituitary adenylate cyclase-activating polypeptide specific receptor, *TGFβ* transforming growth factor β, *TNFα* tumor necrosis factor α, *TSG-6* tumor necrosis factor α-induced protein 6.

A summarized putative schematic diagram of how hMSCs suppressed the effects of SCI is given in Figure 6. (a) Injected hMSCs migrate toward the peri-injury site. (b) There, the hMSCs might be activated or stimulated by inflammatory and/or injury stress-related factors including INFγ. (c) Stimulated hMSCs produce and release human anti-inflammatory factors such as TSG-6, IL-10, TGFβ, and growth factors. (d) Simultaneously, hMSCs and recipient tissues might co-produce IL-4 and induce an AAM environment. (e) Together with reinforcing the anti-inflammatory response, the environment and growth factors from hMSCs induce the production of recipient PACAP, which may then feed back to PAC1R on hMSCs and could further enhance the hMSCs’ rescue signals in a positive manner. (f) Finally, by cross-talk processes, these signals both in the donor hMSCs and in the recipient induce neural rescue and repair at the injury site.

## Additional files

Additional file 1: Figure S1. Expression of mouse *Adcyap1* in hippocampus after transient global ischemia. C57/BL6 mice were subjected to 15 min of common carotid artery occlusion and were injected with hMSCs (1 × 10^5^ cells) or vehicle (HBSS) into each dentate gyrus the next day. One day after cell implantation, the hippocampi were extracted and analyzed by a mouse microarray system. The same procedures were performed in sham-operated animals not subjected to ischemia. DNA microarray data were reanalyzed for our previous work. Additional file 2: Figure S2. Validation of PKH-labeled hMSCs. PKH26-labeled hMSCs were characterized prior to injection. (A) PKH-labeled hMSCs were stained with either anti-CD59 (Beckman) or anti-Human Nuclei (Chemicon) antibodies to validate that the labeled cells were hMSCs. The blue color represents DAPI staining of nuclei. (B) The fluorescence intensity (Ex 544, Em580) of cells was also measured. While non-labeled control cells (cont) did not show any change in signal intensity, labeled cells clearly showed an increased fluorescence signal.

## Abbreviations

AAM: Alternatively activated (activating) macrophage
BDNF: Brain-derived neurotrophic factor
BMS: Basso Mouse Scale
CAM: Classical activating macrophage
CCM: Complete culture medium
CNS: Central nervous system
DAM: Deactivating macrophage
DAPI: 4,6-Diamidine-2-phenylindole dihydrochloride
GFAP: Glial fibrillary acidic protein
HBSS: Hank’s balanced salt solution
hMSCs: Human mesenchymal stem/stromal cells
IFNγ: Interferon-γ
IL-10: Interleukin-10
IL-1β: Interleukin-1β
IL-4: Interleukin-4
MΦ: Macrophage
NGF: Nerve growth factor
NTF3: Neurotrophin 3
PAC1R: Pituitary adenylate cyclase-activating polypeptide specific receptor
PACAP: Pituitary adenylate cyclase-activating polypeptide
PMA: Phorbol myristate acetate
SCI: Spinal cord injury
TGFβ: Transforming growth factor β
TNFα: Tumor necrosis factor α
TSG-6: Tumor necrosis factorα-induced protein 6
VIP: Vasoactive intestinal peptide
WT: Wild-type
