# Supplementary figures and images for: Human mesenchymal stem/stromal cells suppress spinal inflammation in mice with contribution of pituitary adenylate cyclase-activating polypeptide (PACAP)
Source: J Neuroinflammation. 2015 Feb 22;12:35. doi: 10.1186/s12974-015-0252-5 (PMC4346126; doi:10.1186/s12974-015-0252-5)

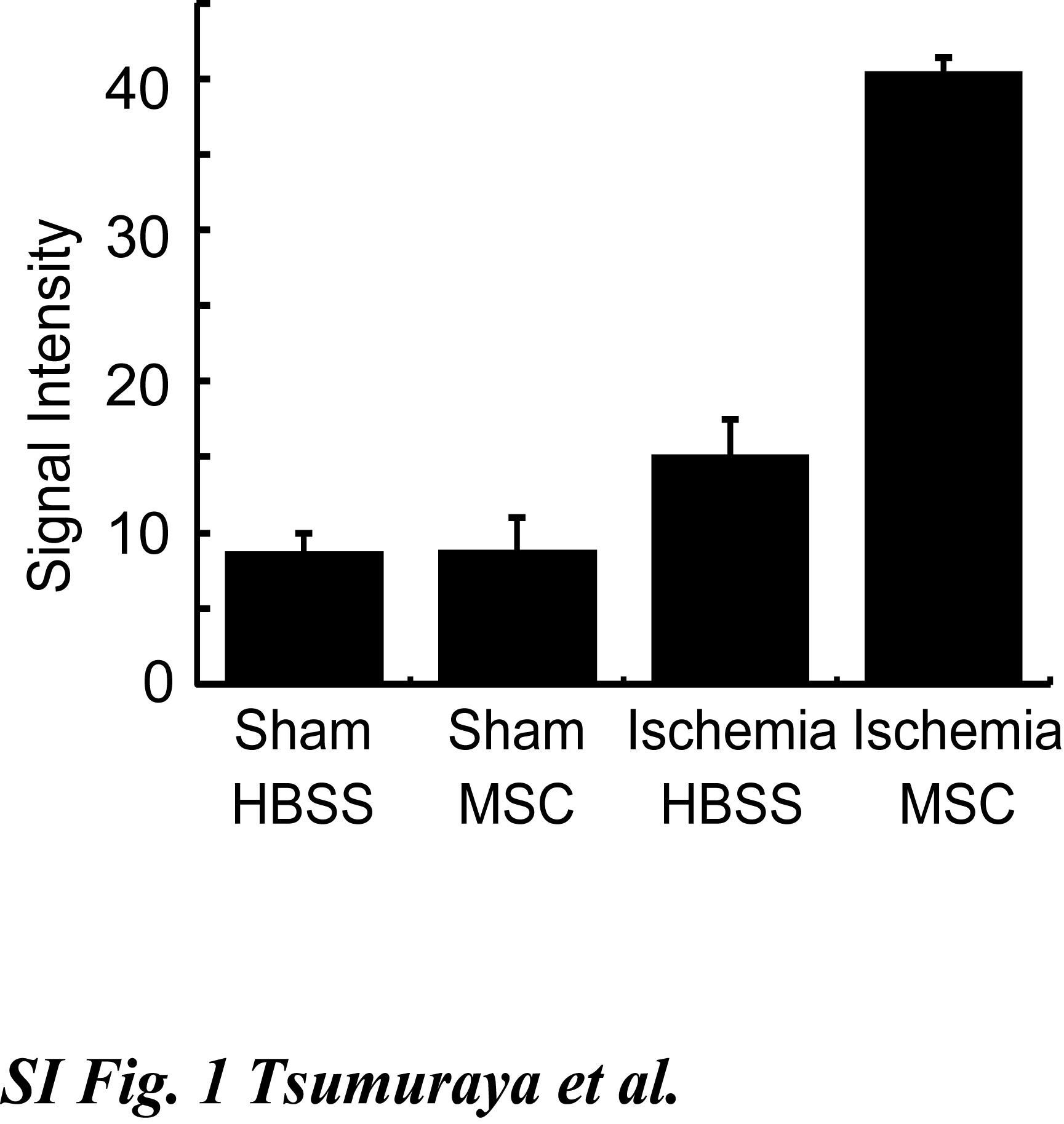

Supplement: Additional file 1: Figure S1. — Expression of mouse Adcyap1 in hippocampus after transient global ischemia. C57/BL6 mice were subjected to 15 min of common carotid artery occlusion and were injected with hMSCs (1 × 10^5^ cells) or vehicle (HBSS) into each dentate gyrus the next day. One day after cell implantation, the hippocampi were extracted and analyzed by a mouse microarray system. The same procedures were performed in sham-operated animals not subjected to ischemia. DNA microarray data were reanalyzed for our previous work. [file 12974_2015_252_MOESM1_ESM.tiff]

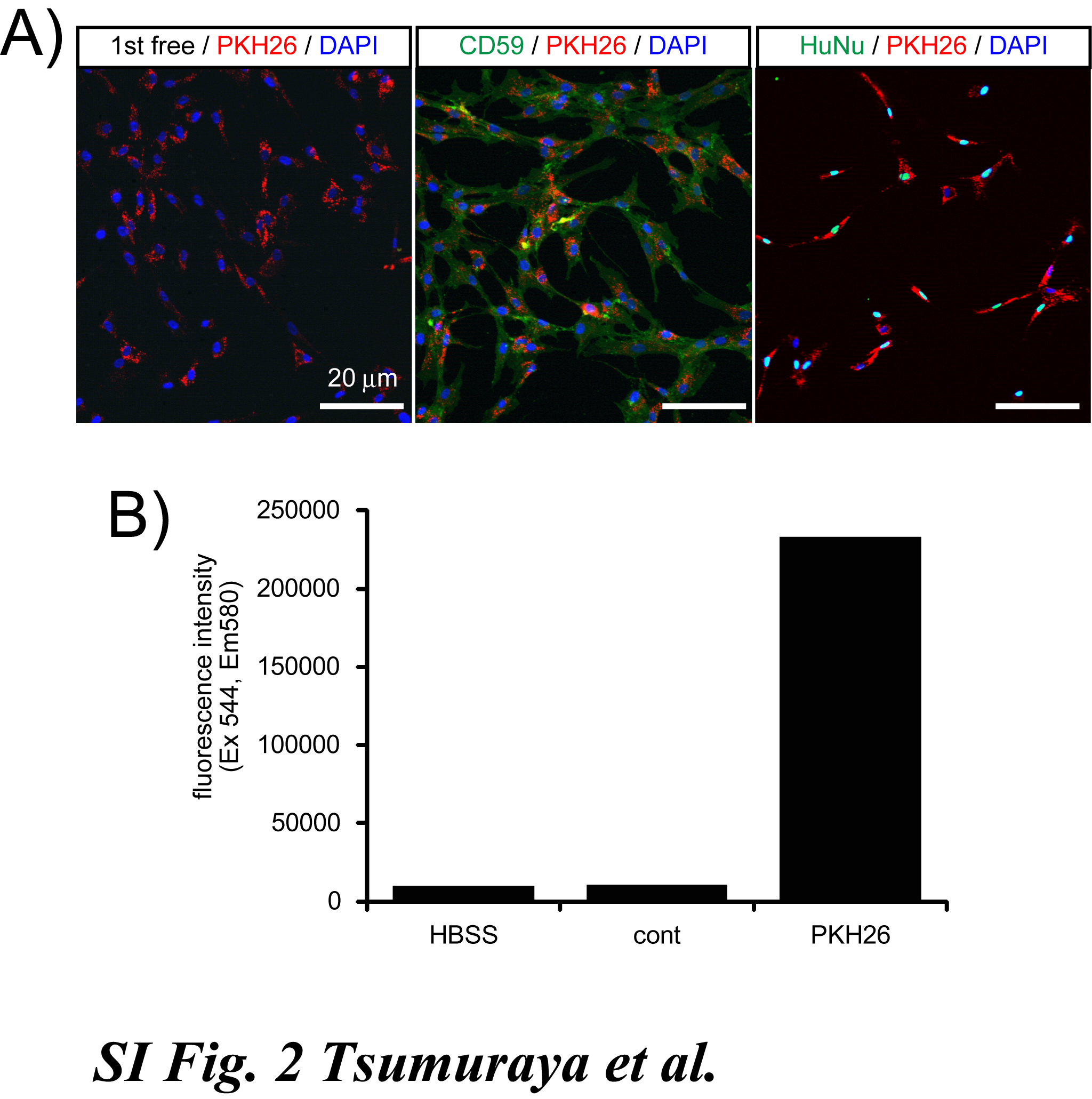

Supplement: Additional file 2: Figure S2. — Validation of PKH-labeled hMSCs. PKH26-labeled hMSCs were characterized prior to injection. (A) PKH-labeled hMSCs were stained with either anti-CD59 (Beckman) or anti-Human Nuclei (Chemicon) antibodies to validate that the labeled cells were hMSCs. The blue color represents DAPI staining of nuclei. (B) The fluorescence intensity (Ex 544, Em580) of cells was also measured. While non-labeled control cells (cont) did not show any change in signal intensity, labeled cells clearly showed an increased fluorescence signal. [file 12974_2015_252_MOESM2_ESM.tiff]
